# Effect of HAc on the Metastable Pitting Corrosion of 304 SS in NaCl Solution

**DOI:** 10.3390/ma15103618

**Published:** 2022-05-18

**Authors:** Hanlu Zhang, Wenqiang Huang, Han Wei, Zilong Chen, Jingyi Cao, Yuming Tang, Xuhui Zhao, Yu Zuo

**Affiliations:** 1 No. 92228 of the People’s Liberation Army, Beijing 100072, China; zhanghanlu_123@163.com; 2 Key Laboratory of Carbon Fiber and Functional Polymers, Ministry of Education, Beijing University of Chemical Technology, Beijing 100029, China; m18810264207@163.com (W.H.); 2019210177@buct.edu.cn (H.W.); 2018020117@buct.edu.cn (Z.C.); xhzhao@mail.buct.edu.cn (X.Z.); zuoy@mail.buct.edu.cn (Y.Z.)

**Keywords:** 304 stainless steel, 0.6 mol/L NaCl, metastable pitting corrosion, acetic acid, pH

## Abstract

Stainless steels (SSs) easily suffer localized corrosion damage, such as pitting corrosion, in mixed solutions of acetic acid and sodium chloride. Currently, few works have been focused on the early stages of the pitting corrosion (metastable pitting corrosion) process of SSs in a chloride-HAc mixture solution. In this work, the effects of acetic acid (HAc) and its concentration on metastable pitting corrosion and the uniform corrosion of 304 SS in 0.6 mol/L NaCl solution were investigated by a slow-scanning potentiodynamic polarization test, scanning electron microscopy (SEM), and X-ray photoelectron spectroscopy (XPS). The results show that the uniform corrosion rate of 304 SS increases after HAc addition but, with an increase in HAc concentration, the corrosion rate decreases. In the presence of HAc, the metastable pitting potential (*E*_m_) and stable pitting potential (*E*_b_) move negatively, but the number of metastable pits notably decreases. HAc has a promoting action on the growth rate of the metastable pits and facilitates the transition from metastable pits to stable pits. The influence of HAc is related to a decrease in solution pH and the chemical adsorption of HAc.

## 1. Introduction

Currently, 304 stainless steel (SS) is commonly used in petrochemical engineering, metallurgy machinery, equipment, electrical hardware and kitchenware due to its good toughness, plasticity, weldability and appreciable corrosion resistance. In petrochemical and kitchenware applications, when acting as a container, the stainless steel sometimes comes in contact with solutions involving organic acids and sodium chloride. Acetic acid is one of the most common organic acids. Stainless steels in a mixed solution of acetic acid and sodium chloride will suffer localized corrosion damage, such as pitting corrosion [1,2].

During the pitting corrosion process of stainless steels, before the occurrence of stable pits, some micro-sized metastable pits may occur, reflected by the transient fluctuations of the current in electrochemical monitoring signals, which correspond to the process of nucleation, growth and repassivation of the metastable pits [3,4]. If the metastable pits grow continuously and do not repassivate, they will develop into stable pits. Pitting corrosion is a stochastic process. Except for the material’s structure and components, some environmental factors, such as solution composition, pH and temperature, also influence on the corrosion process [5]. Chloride ions (Cl^-^) are one of the most important media components; these can adsorb on the surface of stainless steel and react with metal or oxide to form soluble chlorides, resulting in the breakdown of the passive film and the initiation of pitting [6]. The higher the chloride concentration, the higher the nucleation rate of metastable pits and the greater the chance of transition to stable pits [7,8,9]. Gong et al. [10] studied the effect of solution pH on the metastable pitting of 316L SS. The results show that with an increase in pH value, both the metastable pitting potential (*E*_m_) and the stable pitting potential (*E*_b_) move in a positive direction, and the number of metastable pits reduces. Pardo et al. [11] studied the effect of solution pH on the stable pitting behavior of high-alloy SS, and pointed out that the pitting potential shows little tendency to decrease with an increase in pH value.

It is known that acetic acid (HAc) is a weak acid that can partially dissociate into hydrogen and acetate ions (HAc → H^+^ + Ac^−^), thereby decreasing the pH of a solution [1,12,13]. Some investigators have reported that the presence of HAc increases the corrosivity of a solution [13,14], whereas others have reported that HAc works as a weak anodic inhibitor [15]. Among the previous studies published on the corrosion of steels in acetic acid, many were focused on carbon steels. Crolet et al. [15] and Asmara et al. [16] studied the corrosion behaviors of carbon steels in an oil and gas production environment containing H_2_S, CO_2_ and acetic acid. The results show that the presence of acetic acid causes an increase in the corrosion rate because the corrosion product of iron acetate has greater solubility compared with iron bicarbonate. The studies of Talukdar et al. [17] indicate that in a CO_2_-H_2_S solution, at lower concentrations of acetic acid, the corrosion rate of carbon steel increases, while at higher concentrations of acetic acid, the corrosion rate decreases but pitting corrosion is observed. This is because the presence of more H^+^ ions, supplied by HAc, reduces the adsorption of H_2_S species on carbon steel, resulting in a lower anodic dissolution rate. Gulbrandsen et al. [18] studied the effect of acetic acid on the pitting propagation of carbon steel in a NaCl solution with CO_2_. It was shown that at room temperature, with increasing HAc concentration, the corrosion potential increases and the pit propagation rate increases. 

Other authors have also focused on the corrosion behavior of stainless steel in acetic solution. Early on, Sikine and Momoy, as well as Leontaritis et al. [19,20], studied the corrosion behavior of stainless steel in boiling acetic acid solutions and almost anhydrous acetic acid, mixed with chloride ions. In recent years, Wei et al. [21] studied the effect of acetic acid on the pitting corrosion of 2Cr12MoV turbine steel in deoxygenated condensate solution containing chloride ions (90 °C). The results show that acetic acid increases the corrosion rate and decreases the pitting corrosion resistance. Abel et al. [22] investigated the corrosion of martensitic stainless steel in ethanol-containing gasoline mixtures and found that a higher amount of acetic acid leads to higher pit density but has little effect on pit propagation in terms of depth. Li et al. [23] investigated the corrosion behavior of 316L SS in oilfield-produced water in the presence of CO_2_ and acetic acid. The results show that with an increase in acetic acid concentration, the corrosion rate of 316L SS first increases and then decreases. Acetic acid at a low concentration could damage the passive film. Mahato et al. [1] studied the pitting behavior of food-grade ferritic AISI-430 SS in 20% acetic acid with 0.5 mol/L NaCl and demonstrated that the acetate ions in the solution promote the dissolution of Fe and Cr atoms. Their results also showed that potentiodynamic polarization measurements at a slow scanning rate revealed the formation of metastable pits and repassivation. 

Previous works have proposed that the metastable pitting stage represents the early stage of pitting corrosion and that there is a close relationship between metastable and stable pitting behaviors [5,6]. Therefore, understanding the mechanism of metastable pitting is helpful for predicting the pitting tendencies of metals [4,7]. So far, in terms of the published papers studying the pitting corrosion of 304 SS in chloride-HAc mixture solutions, very few of them paid attention to the metastable pitting corrosion process. In this work, the behaviors of pitting corrosion, including metastable pitting and stable pitting, and the uniform corrosion of 304 SS in 0.6 mol/L NaCl with different concentrations of acetic acid were studied in slow-scanning potentiodynamic polarization tests. Combined with scanning electron microscopy (SEM) and X-ray photoelectron spectroscopy (XPS) measurements, the influences and mechanisms of acetic acid and the pH of solutions in terms of the nucleation and growth processes of metastable pits were analyzed.

## 2. Materials and Methods

The test material used was 304 stainless steel (304 SS) with a chemical composition of (wt %): C 0.035, Si 0.52, Mn 1.18, P 0.036, S 0.026, Cr 17.59, Ni 8.03 and Fe 72.6. The size of the specimen was 1 cm × 1 cm × 1 cm. The working surface of the specimen was abraded with 400, 600, 800 and 1000 grit silicon carbide paper, successively, and then cleaned with deionized water and alcohol. After a copper wire was welded to one end, the specimen was coated with 704 silicon rubber, leaving an area of 0.16 cm^2^ exposed to the test solution. 

The basic solution comprised 0.6 mol/L NaCl solution (pH 7.2). In order to study the effect of HAc concentration on the corrosion behavior of 304 SS, 0.8 mol/L, 1.6 mol/L and 3.2 mol/L acetic acid (HAc), respectively, was added into the basic solution. The pH values of the solutions were measured with a pH meter (PHS-25 style, Shanghai, China), which gave readings of 2.3, 2.2 and 2.1, respectively. In order to investigate the influence of solution pH on the 304 SS corrosion, the pH value of the basic solution was adjusted to 2.3 using the hydrochloric acid titration method. During the titration process, the content of Cl^-^ ions in the droplet was calculated and the NaCl concentration in the basic solution was reduced accordingly so that the total content of Cl^-^ ions was maintained unchanged at 0.6 mol/L.

The slow-scanning potentiodynamic polarization curves of SS specimens in the above solutions were measured using a CS350 electrochemical workstation (Corrtest Company, Wuhan, China). The polarization test started at a potential 250 mV below the open-circuit potential, at a potential scanning rate of 0.1 mV/s in the anodic direction, until the stable pits occurred. A three-electrode system was applied in the test. The reference electrode was a saturated calomel electrode (SCE), the counter electrode was a platinum electrode and the working electrode was a 304 SS specimen. All the tests were carried out at ambient temperature. Because of the stochastic characteristic of pitting corrosion under each experimental condition, at least five tests were run. Then, the data from five or more parallel tests were statistically analyzed.

The potential when the first apparent current fluctuation occurred, with a maximum peak higher than 0.02 μA, was denoted as the metastable pitting potential (*E*_m_), and the potential when the current increased continually was denoted as the stable pitting potential (*E*_b_). The rate of metastable pit initiation was obtained by the accumulative quantity of the current fluctuations [3,24]. Each current peak displays the typical characteristic of a slow rise followed by a quick drop. The slope of the rise stage represents the average growth rate of the metastable pit (*K* = *I*_peak_/*t*_grow_), where *I*_peak_ is the peak current and *t*_grow_ is the growth time of a metastable pit. 

Potential step tests were also performed for the 304 SS specimens. During the test, the potential of the specimen was stepped up from −500 mV_SCE_ to −180 mV_SCE_ quickly; meanwhile, the current transient value was recorded. The action of acetic acid on the active–passive transition of the film was discussed.

After electrochemical measurement, the surface morphology of the specimen was observed with a scanning electron microscope (XL30 ESEM, Philips, Amsterdam, The Netherlands), while the elements were analyzed with an energy disperse spectroscope (EDS) (NORAN Vantage, Thermo Scientific, Waltham, MA, USA). The elemental composition and chemical valence of the film on the 304 SS were analyzed with a X-ray photoelectron spectroscopy (XPS) (ESCALAB 250, Thermo VG, Waltham, MA, USA). The spectra were fitted using Avantage software (V 5.948, Waltham, MA, USA).

## 3. Results and Discussion

### 3.1. Corrosion Behavior of 304 SS in 0.6 mol/L NaCl Solutions with HAc

**Figure 1:**
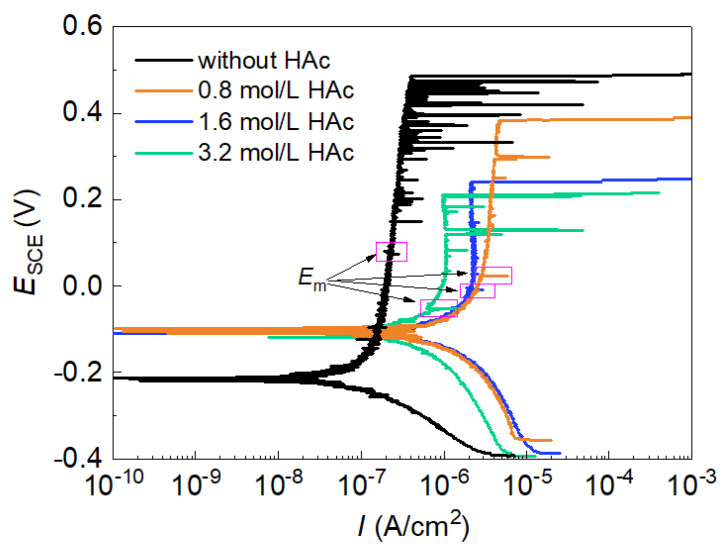
Polarization curves of 304 SS in 0.6 mol/L NaCl solutions with different concentrations of HAc.

Figure 1 shows the potentiodynamic polarization curves of 304 SS in 0.6 mol/L NaCl solutions, both without and with 0.8 mol/L, 1.6 mol/L or 3.2 mol/L of HAc. The polarization curve in the basic solution (0.6 mol/L NaCl solution, pH 7.2) presented a broad passive region with a very low passive current density (0.35 μA/cm^2^). As the potential increased, many current fluctuations were observed in the passive region, which indicated the occurrences of metastable pitting corrosion before stable pitting occurred [3,24]. After HAc was added, the corrosion potential of 304 SS moved in a positive direction, the broadness of the passive region was reduced, the passive current density visibly increased and the number of current fluctuations was reduced significantly. These findings demonstrate the decreasing passivity of the passive film. It was noted that after HAc addition, the pH value of the solution changed from 7.2 to 2.3, 2.2 and 2.1, respectively. Therefore the decreased passivity of 304 SS is probably related to the combined influence of solution pH and acetate ions. 

The parameters of the polarization curves in each condition were statistically analyzed. Table 1 shows the average values of the parameters, in which the corrosion current density (*I*_corr_) was obtained using the Tafel extrapolation method, performed on the cathodic branch. It can be seen that after the addition of 0.8 mol/L HAc, the corrosion potential (*E*_corr_) moved visibly in a positive direction, while both the corrosion current density (*I*_corr_) and the passive current density (*I*_pass_) increased notably, manifesting a decreased passivity of SS and an enhanced film dissolution rate. However, as the concentration of HAc increased, both *I*_corr_ and *I*_pass_ presented a decreasing tendency, but this was still much higher than that obtained in the solution without HAc. The positive shift of the corrosion potential is probably caused by an inhibition of the anodic reaction and an increase in the cathodic reaction rates [14,18]. Moreover, the addition of HAc caused both the metastable pitting corrosion potential (*E*_m_) and the stable pitting corrosion potential (*E*_b_) to move negatively; with HAc concentration increasing, *E*_m_ and *E*_b_ moved further toward a negative direction. Generally, the parameter *R*_pit_ (= *E*_b_ − *E*_corr_) can be used to evaluate the pitting resistance (or sensitivity), and a smaller *R*_pit_ value means higher pitting susceptibility [25]. The data in Table 1 demonstrate that the pitting susceptibility of 304 SS increases with the increase in HAc concentration. Therefore, HAc has a promotion effect on the occurrence of both metastable pits and stable pits.

**Figure 2:**
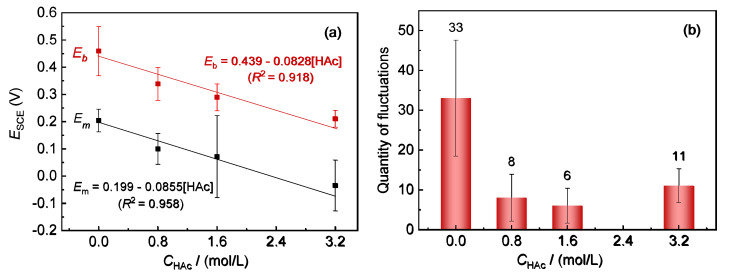
Variation of *E*_m_ and *E*_b_ of 304 SS with different HAc concentrations, in 0.6 mol/L NaCl solution (**a**) and a bar graph of the number of current fluctuations of 304 SS in 0.6 mol/L NaCl solutions with HAc (**b**).

Figure 2a shows the variations in *E*_m_ and *E*_b_ of 304 SS with HAc concentration in 0.6 mol/L NaCl solution. With an increase in the HAc concentration, both *E*_m_ and *E*_b_ present a similar decreasing tendency. When fitting the data of *E*_m_ and *E*_b_ with HAc concentration using the Origin software (Origin 2018, Northampton, MA, USA), the results show that all values changed linearly with HAc concentration (*E* = a × *C*_HAc_ + b). This means that there was a very good correlation between *E*_m_ and *E*_b_, which is similar to the results for *E*_m_ and *E*_b_ with the variation of Cl^-^ concentration in a previous work [24]. 

The nucleation rate of metastable pits can be reflected by the number of current fluctuations in the polarization curves [3,24]. Figure 2b shows the average values of the accumulative quantity of current fluctuations in five parallel testing curves for 304 SS in each solution. After the addition of HAc, the number of current fluctuations is reduced significantly, from the initial number of 33 to an average value of about 9 (6–11), with the addition of 0.8 mol/L to 3.2 mol/L HAc. These indicate that the presence of HAc has a notable inhibitory action on the nucleation numbers of the metastable pits, which might be associated with the competitive adsorption between acetic acid and chloride ions on the active sites of the steel’s surface.

**Figure 3:**
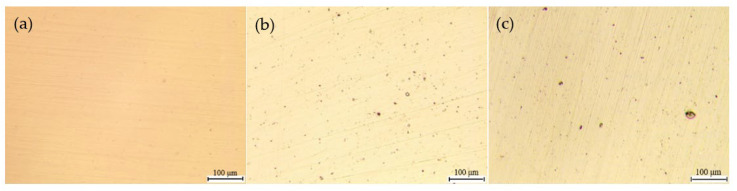
Optical micrographs (100 times) of 304 SS: (**a**) before polarization, (**b**) after polarization in 0.6 mol/L NaCl solution, (**c**) after polarization in 0.6 mol/L NaCl solution with 3.2 mol/L HAc.

**Figure 4:**
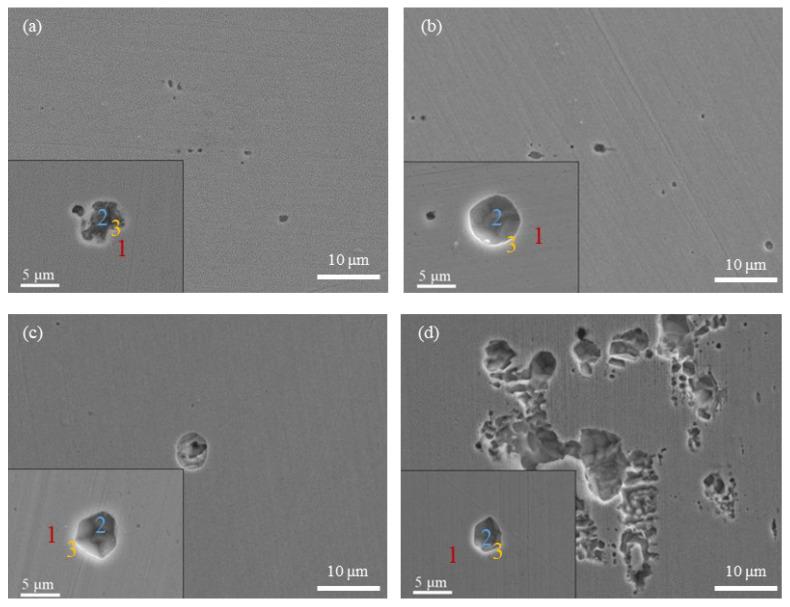
SEM images of 304 SS after polarization in an 0.6 mol/L NaCl solution, with different concentrations of HAc: (**a**) without HAc, (**b**) 0.8 mol/L HAc, (**c**) 1.6 mol/L HAc, (**d**) 3.2 mol/L HAc.

Figure 3 presents the optical micrographs (100 times) of the specimens after potentiodynamic polarization tests. In the basic solution, many small pits were observed, with sizes mostly in the range of 2.5 μm–5 μm (Figure 3b). In the presence of HAc, the number of small pits was markedly reduced, corresponding to the reduction in the number of current fluctuations in the polarization curves. Figure 3c shows the surface morphology of the specimen in the presence of 3.2 mol/L HAc, in which several pits with diameters larger than 10 μm are observed. Figure 4 shows the corrosion morphologies using SEM. It can be clearly seen that after HAc addition, the number of metastable pits was reduced, while the dimensions of the pits increased. In Figure 4d, with the addition of 3.2 mol/L HAc, some pits connected up with each other, forming a large area of corrosion damage.

The EDS method was used to analyze the chemical composition of the corroded areas of 304 SS under each set of conditions. The analyzed areas include the ones on the steel matrix (Area 1), the ones inside the pits (Area 2) and the ones on the edges of the pits (Area 3), which are marked in a locally enlarged view of each image in Figure 4. The EDS results are shown in Table 2. It can be seen that without HAc addition, the element contents of sulfur (S) and manganese (Mn), both inside and on the edge of the pits, are higher than those on the surface of the steel (matrix), which indicates that inclusions containing S and Mn may be the active sites for metastable pitting. This is in good agreement with previous studies, which reported that MnS inclusions are preferential sites for metastable pitting events in stainless steel [24,26]. With the addition of HAc, the content of the Mn element shows a decreasing tendency; meanwhile, the S contents, both inside and outside the pits, decreased even as far as zero. This is probably because the presence of the HAc accelerates the dissolution of the steel surface and the inside of the pits. When the pits are relatively deep, the MnS inclusions are more prone to dissolve or are etched off into the solution. Zheng et al. [27] investigated the variation in inclusions on 2205 duplex SS after being corroded with 3.5% NaCl solution and reported similar results. They pointed out that when the corrosion rate increases, MnS inclusions are more easily dissolved. Hence, the decrease in the S and Mn contents inside and on the edges of the pits when in the presence of HAc could also reflect the fact that HAc promotes the growth of metastable pits on 304 SS.

**Figure 5:**
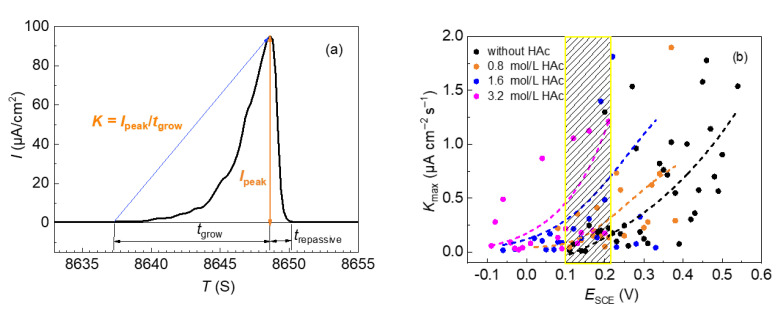
Typical current fluctuation of 304 SS in 0.6 mol/L NaCl solution, using a potentiodynamic polarization test (**a**) and average growth rate of metastable pits on 304 SS, in 0.6 mol/L NaCl solutions with HAc (**b**).

The average growth rate of the metastable pits of 304 SS in 0.6 mol/L NaCl solutions with different concentrations of HAc was statistically analyzed. Figure 5a shows a typical current transit fluctuation of 304 SS in the slow-scanning potentiodynamic polarization test, including the annotation of the parameters. It can be seen that the growth rate of the metastable pit can be divided into two stages, the first, shorter, stage of which demonstrates a slower rate, while the second, longer, stage shows a quicker rate. In this work, the average growth rate in the second stage of each current fluctuation was used to represent the average growth rate of a metastable pit. Because the five parallel tests produced many data points, the entire potential region involving all current fluctuations was divided into several sections, using 10 mV as an interval. In each potential section, the biggest average growth rate (*K*_max_) of the metastable pit was selected and the influence of HAc concentration was analyzed. Figure 5b presents the largest average growth rate (*K*_max_) in the different potential sections. It can be seen that the values of *K*_max_ are mainly in the range of 0–2.0 μA cm^−2^/s. In order to show the comparisons more clearly, the trend of the data points was fitted under each solution condition, which is shown by the dotted line. At the same potential (the shaded area in Figure 5b), the value of *K*_max_ increased with increasing HAc concentration. This demonstrates that the presence of HAc has a promoting action on the growth rate of metastable pits on 304 SS.

**Figure 6:**
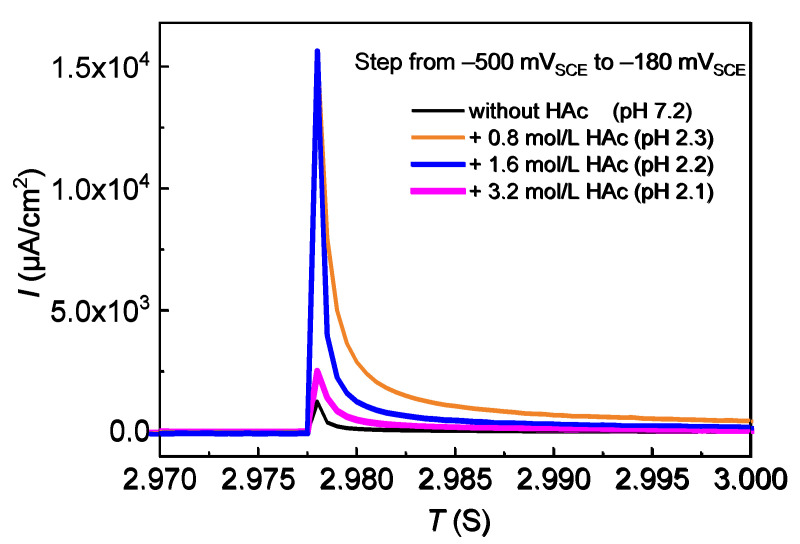
Current time-curves of 304 SS in 0.6 mol/L NaCl solutions with HAc during the step tests of the potential.

The potential step tests were performed, using 304 SS and 0.6 mol/L NaCl solutions with HAc additions, in order to analyze the effect of HAc on the passivity ability of 304 SS. In the potential step process, the current density maximum and the current decay speed are closely related to the passivity ability of the specimen [7]. Using the potentiodynamic polarization curves, the potential was selected to cause a sudden step from −500 mV_SCE_ (in active range) to −180 mV_SCE_ (in passive range), and the current-time curve was recorded. The results are shown in Figure 6. It can be seen that the current maximum and the decay speed are visibly influenced by HAc concentration. In tests, 304 SS presented the lowest current maximum and the shortest time of transition from an active state to a passive state with 0.6 mol/L NaCl solution, which demonstrates that this makes it easier for the 304 SS to reach a passive state. In the presence of 0.8 mol/L HAc, the maximum current density increases remarkably, and the time needed for passive film formation is much longer, which means a faster initial dissolution rate and a slower rate of forming a passive film in the presence of HAc. This is probably due to the decrease in pH value of the solution from 7.2 to 2.3. As the HAc concentration increases to 1.6 mol/L, the current maximum shows no major change compared to that with 0.8 mol/L HAc, but the surface passivity rate is increased. When the HAc concentration increased to 3.2 mol/L, the current maximum and the time for passive film formation all decreased, which demonstrates a decreased dissolution rate of 304 SS and an increased passivity ability with increasing HAc concentration. The results of the potential step tests are in good agreement with those of the potentiodynamic polarization tests.

The above results indicate that the presence of a certain amount of HAc in the 0.6 mol/L NaCl solution (pH 7.2) has obvious promoting effects on the uniform corrosion of 304 SS. This could be due to the presence of Hac, causing the pH of the solution to decrease significantly from a neutral (pH 7.2) to an acid level (pH 2.3–2.1) because of the dissociation reaction (HAc → H^+^ + Ac^−^). In the acid solution, numerous hydrogen ions (H^+^) participate in the electron reaction, instead of the limited amount of oxygen molecules (O_2_) available to electrons in a neutral solution; hence, the cathodic reaction rate increases, thereby increasing the corrosion rate significantly. In addition, as a weak acid, acetic acid is only partially dissociated in an aqueous solution. Hence, both acetate ions (Ac^−^) and undissociated acetic acid (molecular HAc) are present in the solution. Based on the calculation in Equation (1) of the dissociation constant (*K*_Hac_) and the value of the acetic acid (1.753 × 10^−5^ at 25 °C) [1,13], the concentration of the dissociated acetate ions (Ac^−^), the undissociated acetic acid (Hac) and the hydrogen ions (H^+^) were calculated in each solution; the results are shown in Table 3. It can be seen that under these conditions, acetic acid mainly exists in the form of molecules; there are very few dissociated acetate ions (0.00243 mol/L–0.00703 mol/L), which can, basically, be ignored. With the concentration of Hac increasing from 0.8 mol/L to 3.2 mol/L, the number of H^+^ ions in the solutions changed from 10^−2.3^ to 10^−2.1^, with little variation, while the amounts of undissociated acetic acid increased by approximately double and quadruple. It was reported that acetic acid could be chemically adsorbed by the metal surface and had an inhibitive effect on the iron dissolution reaction [15,28,29,30]. Therefore, more acetic acid led to a higher inhibitive effect. Thus, this may explain why the corrosion rate of 304 SS decreased with the increasing Hac concentration in 3.5% NaCl solution, since passivation is easier to achieve under these conditions.

(1) $$K_{\mathrm{HAc}}=\frac{\left[Ac^{-}]\times [H^{+}\right]}{\left[\mathrm{HAc}\right]}$$

**Figure 7:**
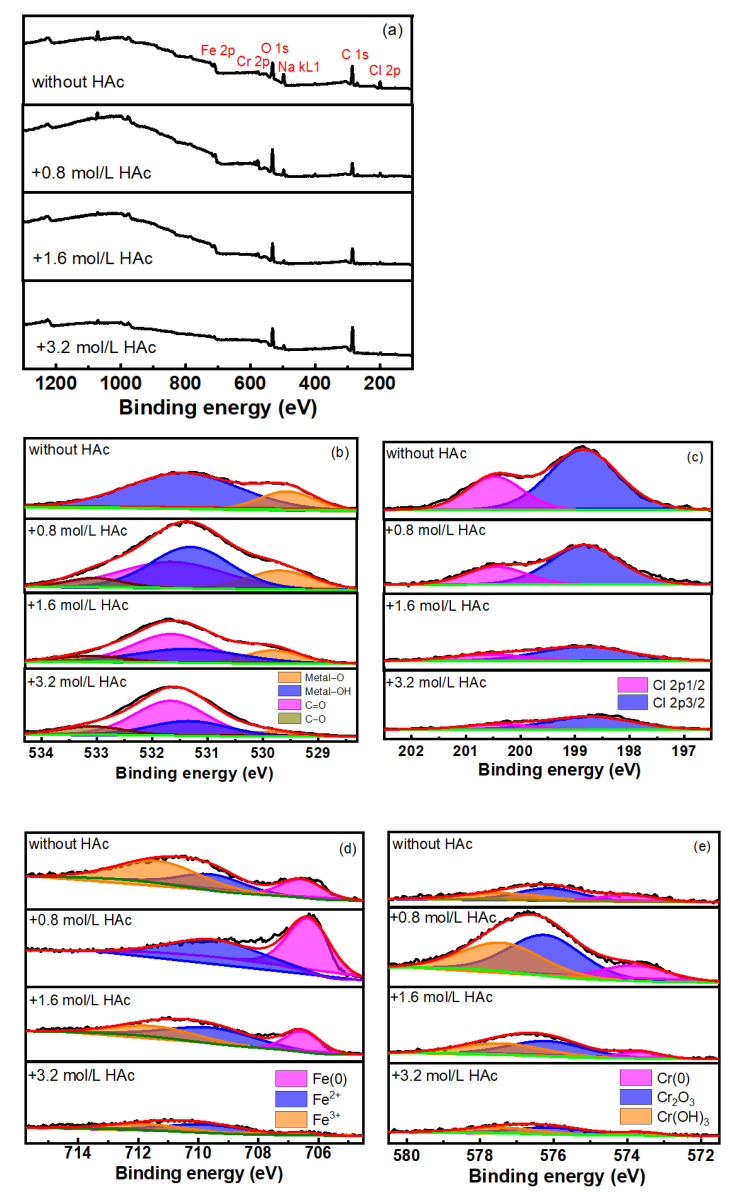
Peak fitting results of spectra of the surface film on 304 SS in 0.6 mol/L NaCl solution with HAc. (**a**) full spectra; (**b**) O 1s; (**c**) Cl 2p; (**d**) Fe 2p3/2; (**e**) Cr 2p3/2.

In order to understand the effect of HAc on the composition of the passive film and the passivity of 304 SS, an XPS test was performed on 304 SS after immersion in various 0.6 mol/L NaCl solutions with different concentrations of HAc. Figure 7a shows the full spectrum of the XPS. It indicates that C, O, Fe, Cr, Cl and Na elements are present in the passive film. Because of the possible presence of contaminants, the spectrum of C 1s was not analyzed [31]. Figure 7b–e show the high-resolution spectra of O1s, Cl2p, Fe2p_3/2_ and Cr2p_3/2_. In Figure 7b, under basic solution conditions, two characteristic peaks are obtained at binding energies of 530 eV and 531.3 eV, corresponding to metal oxides (metal-O) and hydroxides (metal-OH), respectively [32,33,34]. After the addition of HAc, two new characteristic peaks at 531.6 eV and 533.1 eV appeared, corresponding to the C-O and C=O bonds [35,36]. Because the molecular structure of acetate contains both C-O and C=O bonds, the existence of these two peaks indicates the presence of acetic acid or acetate ions adsorbed on the surface of the steel. Due to the acetic acid dissociation equilibrium, HAc mainly existed in a molecular form in the solutions (Table 3). Therefore, the adsorption on the surface of the 304 SS might mainly be due to the acetic acid molecules.

In Figure 7c, the Cl 2p spectrum is decomposed into two peaks, these being the Cl 2p_1/2_ and Cl 2p_3/2_ peaks. The addition of HAc led to an obvious weakening of both peak intensities and, as the HAc concentration increased, the peak intensities decreased. This might be due to the competitive adsorption of HAc by chloride ions (Cl^-^) and the subsequent removal of some Cl^-^ ions from the surface of the stainless steel. It can also be assumed that the amount of HAc adsorbed increased with the increase in HAc concentration. Figure 7d presents the peak fitting results of the Fe 2p_2/3_ spectra. The three peaks, at binding energies of 706.7 eV, 709.5 eV and 711.1 eV, corresponding to the metallic Fe, Fe(II) and Fe(III) valance states [31,32,33,34,35,37,38]. Generally, the passive film found on stainless steel has a bilayer structure, which is composed of an inner chromium oxide-rich layer and an external layer, rich in hydroxide of iron and chromium combined with iron oxide [39,40,41]. The corrosion resistance of the passive film is closely related to the amounts and valence states of iron and chromium; it is also related to the ratio of Fe^3+^ to Fe^2+^. The higher the Fe^3+^/Fe^2+^ ratio, the higher the amount of Fe_2_O_3_ and FeOOH in the film and the more stable the passive film becomes, which then has better protective properties [39,42]. The ratio of Fe^3+^/Fe^2+^ in the film was calculated from the integrity of the peak area in the XPS spectra, as shown in Table 4. After the addition of HAc, the Fe^3+^/Fe^2+^ ratio was reduced significantly. When the HAc concentration was at 0.8 mol/L, almost no Fe^3+^ could be detected, which is probably related to the decreasing pH of the solution from 7.2 to 2.3 after the HAc was added. Because Fe^3+^ species become unstable in an acid solution [38,41,42], the ferric iron compound in the outer layer is thus vulnerable to being dissolved [43]; consequently, the stability of the passive film is then decreased. However, as the HAc concentration increased, the pH value of the solution experienced very little change (2.3, 2.2 and 2.1), and the ratio of Fe^3+^/Fe^2+^ in the film increased slightly. This indicates that a higher concentration of HAc might have certain promoting effects on the transition of Fe^2+^ to Fe^3+^ in the passive film. 

It also can be seen from Figure 7d that with the addition of 0.8 mol/L of HAc, the peak intensity of metallic iron (706.7 eV) detected in the film is very strong, which implies that the passive film that formed on the surface would be relatively thin [41,43,44]. Figure 7e presents the peak fitting results of the Cr2p_3/2_ spectra, in which three characteristic peaks correspond to metallic Cr (574 eV), Cr_2_O_3_ (576 eV) and Cr(OH)_3_ (577.3 eV), respectively [34,38,39,40]. After the addition of 0.8 mol/L of HAc, the peak intensities of Cr oxides and hydroxides in the film are relatively stronger, which is probably because in an acid solution, the solubilities of Fe oxides are higher than those of Cr oxides, and the ferric oxide in the outer layer film is dissolved preferentially [39,42]. This also confirms why almost no Fe^3+^ could be detected on the surface of the steel. It is generally believed that the presence of Cr_2_O_3_ is helpful in enhancing the corrosion resistance of a passive film [29,33]. The Cr_2_O_3_/Cr(OH)_3_ ratio in the passive film was calculated from the XPS spectra and is shown in Table 4. After the addition of HAc, the ratio of Cr_2_O_3_/Cr(OH)_3_ decreases slightly, meaning a worse corrosion resistance of the film [33,39]. This is in good agreement with the electrochemical test results. The variations in HAc concentration show no major influence on the Cr_2_O_3_/Cr(OH)_3_ ratio, which might be related to the small pH variation in the studied HAc concentrations.

To summarize, the addition of HAc in 0.6 mol/L NaCl solution causes a decrease in the ratios of Fe^3+^/Fe^2+^ and Cr_2_O_3_/Cr(OH)_3_, thereby decreasing the stability of the passive film. This should be related to the solution pH decreasing from neutral (7.2) to an acid level (2.3–2.1) in the presence of HAc. As the HAc concentration increased from 0.8 mol/L to 3.2 mol/L, the ratio of Cr_2_O_3_/Cr(OH)_3_ showed no major change and the Fe^3+^/Fe^2+^ ratio in the passive film increased slightly. Because the oxide and hydroxide of iron mainly existed in the external layer of the passive film, the slight increase of the Fe^3+^/Fe^2+^ ratio may have contributed to improving the corrosion resistance of the 304 SS to a certain degree. The results of the XPS also confirmed the adsorption of acetic acid on the surface of stainless steel and the probable competitive adsorption between acetic acid and Cl^-^ ions, which have an inhibitive effect on stainless steel [28,30]. The XPS results are consistent with those of the polarization and potential step tests.

From the above results, it can be seen that adding HAc to a NaCl solution has various effects on the uniform corrosion and pitting corrosion of 304 SS. After the addition of HAc, the pH of the solution decreases to an acid level, which promotes the dissolution rate of 304 SS. However, when the HAc concentration increases, the corrosion rate does not continuously increase but instead decreases slightly, which is probably related to the adsorption of HAc on the surface of 304 SS, presenting a weak inhibition effect. The effect of HAc on the pitting corrosion of 304 SS can be presented as follows. On the one hand, after HAc addition, the metastable pits and stable pits occur at relatively negative potentials (with both *E*_m_ and *E*_b_ decreasing), the growth of metastable pits is promoted and deeper pits are obtained. On the other hand, the number of metastable pits is reduced. This is very different from the effect of Cl^-^ ions on the pitting corrosion of metals. Generally, Cl^-^ ions cause *E*_m_ and *E*_b_ to decrease, as well as increase both the number and the growth rate of metastable pits for steels or Al alloys [3,4,6,7]. Gulbrandsen et al. [14] investigated the corrosion behavior of X65 pipeline steel when in solutions containing CO_2_, HAc and NaCl. They also found that in the presence of HAc, deep pits developed but the number of pits reduced, which is similar to our findings. This is probably because although the pH of the solution decreased to an acid value in the presence of HAc, the 304 SS sample still presented good passive behavior in a wide potential region. Once attacked by aggressive chloride ions, a few pits were nucleated, which can depolarize the electrode to a potential below the pit nucleation potential [14]. The nucleated pits are in the anodic area and the other areas on the surface may be cathodically protected [45]. Thus, the existing pits continue to grow, causing attacks on a deep level. In addition, according to the results of the XPS, in presence of Hac, the ratios of Fe^3+^/Fe^2+^ and Cr_2_O_3_/Cr(OH)_3_ in the passive film were reduced. Meanwhile, according to the literature [43,46,47], under acid conditions, a relatively thinner and less protective passive film is formed on the surface of stainless steel; hence, upon the action of aggressive chloride ions, the passive film is easily broken down to a lower pit potential. In addition, the acetic acid molecules can be chemically adsorbed on the steel surface and have an inhibitive effect on the anodic and cathodic charge transfer rates [15,28,29,30]. The decreased number of metastable pits in the presence of HAc is probably associated with the competitive adsorption between acetic acid and chloride ions, which may reduce the adsorption number of the aggressive chloride ions on the active sites, thereby reducing the initiation number of metastable pits caused by the dissolution of active sites [28,29,30]. 

### 3.2. Corrosion Behavior of 304 SS in 0.6 mol/L NaCl Solutions with different pH

**Figure 8:**
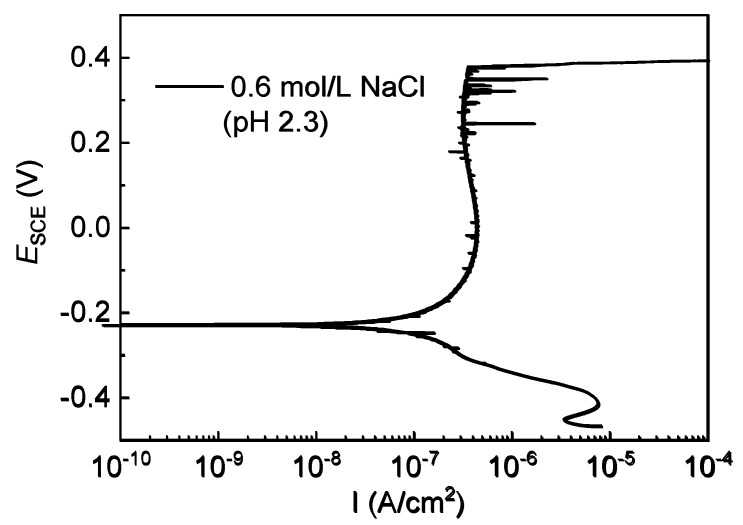
Potentiodynamic polarization curves of 304 SS in 0.6 mol/L NaCl solution (pH 2.3).

In this work, after 0.8 mol/L of HAc was added to 0.6 mol/L of NaCl solution, the pH value decreased significantly from 7.2 to 2.3. As a weak acid, acetic acid is partially dissociated in solution, which means that it not only releases hydrogen ions, lowering the pH, but also supplies plenty of undissociated acetic acid molecules [12,13,30]. In order to understand the effects of solution pH and HAc, the polarization curves were measured for 304 SS in acidic 0.6 mol/L NaCl solution (pH 2.3). Figure 8 shows the typical polarization curve, in which 304 SS still presents good anodic passive behavior. The parameters in the polarization curve were compared with those in the basic solution (pH 7.2). The results in Table 5 show that in the acidic 0.6 mol/L NaCl solution, both corrosion current density (*I*_corr_) and passive current density (*I*_pass_) apparently increase, but the change in the corrosion potential (*E*_corr_) is very small, with just a slight shift to the negative direction, which is in agreement with the findings reported in the literature [44]. This indicates that the decrease in pH accelerates the dissolution of the passive film on 304 SS by supplying hydrogen ions (H^+^). In the acid NaCl solution (pH 2.3), both the metastable pitting potential (*E*_m_) and the stable pitting potential (*E*_b_) moved to a negative direction, demonstrating that the acidic medium promoted the nucleation of both metastable pits and stable pits. This is consistent with the results for 316L SS and high-strength pipeline steel in NaCl solution with pH variations in the literature [10,44], which was explained by the fact that in an acidic solution, the film is relatively thin and is non-uniform so that the passivity of SS is easily damaged and pitting corrosion is thereby initiated in the action of aggressive chloride ions. Generally, the value of the pitting potentials difference (*E*_b_-*E*_m_) can be used to characterize the probability of transition from metastable pits to stable pits. The smaller value means a higher transition probability [9,24]. Table 5 also shows that when the pH value decreased, the difference between *E*_b_ and *E*_m_ significantly decreased, which indicates that in an acidic NaCl solution, the metastable pits transformed easily into stable pits. Based on the occluded corrosion cell theory [48], the lower pH environment in the pits’ interior is more favorable for pitting growth than the pitting repair process, so in acid conditions, the metastable pits are easily propagated and turn into stable pits.

**Figure 9:**
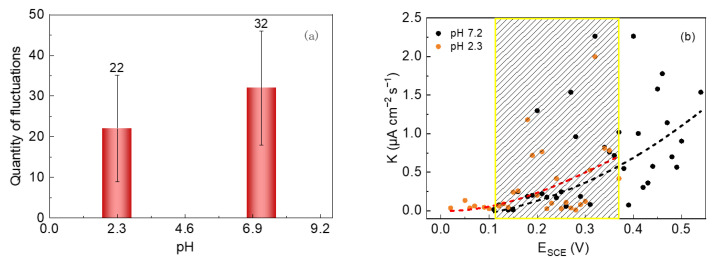
The number of current fluctuations (**a**) and the average growth rate of metastable pits (**b**) of 304 SS in 0.6 mol/L NaCl solutions (pH 2.3 and 7.2).

The nucleation numbers of metastable pits and the average growth rates of 304 SS in the 0.6 mol/L NaCl solutions with pHs of 7.2 and 2.3 were comparatively analyzed, and the results are shown in Figure 9. It can be seen in Figure 9a that the number of metastable pits of 304 SS in the pH 7.2 solution was 32, while it decreased to 22 in the pH 2.3 solution. Obviously, the nucleation number in the acidic solution is much lower, which is probably because the dissolution rate of the passive film is relatively higher in acid conditions; thus, the behavior of the pits with higher electrochemical activities can be recorded, while the other pits with lower activity levels may not be recorded during the entire film dissolution process. In Figure 9b, in the same potential region (the dotted box), as the pH decreased, the average growth rate of metastable pits increased slightly. This is probably because the surface film on stainless steel in an acidic solution is relatively thinner and has poorer stability [43,44]. In addition, compared with a neutral solution, in acidic conditions, there are more hydrogen ions (H^+^) that can migrate into the metastable pits and accelerate the dissolution of metal inside the pits causing the earlier pitting, which cannot be repaired through an autocatalytic process. Thus, the acid solution leads to a greater pitting attack compared to the neutral solution.

**Figure 10:**
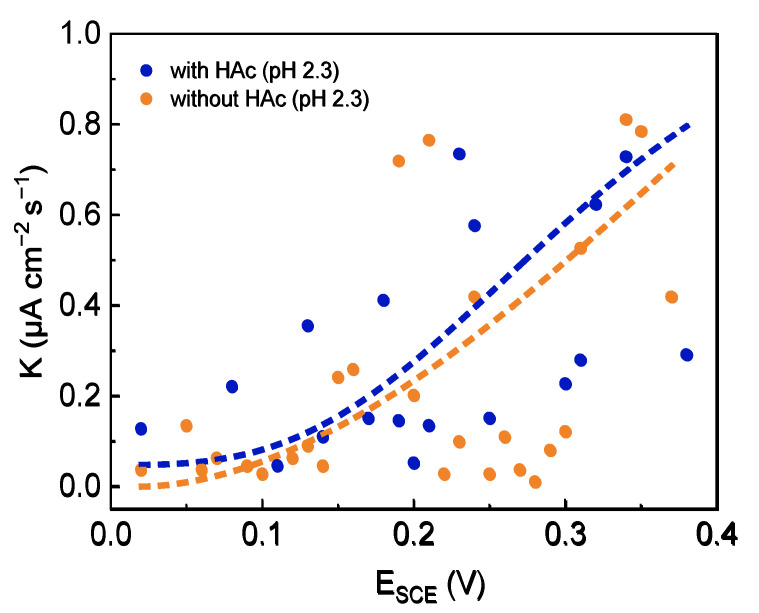
Comparison of the growth rate of metastable pits of 304 SS in 0.6 mol/L NaCl solutions (pH 2.3), both without and with 0.8 mol/L HAc.

In order to analyze the effect of HAc on the metastable pitting behaviors of 304 SS in acid solution, the polarization curves and the related parameters of 304 SS in 0.6 mol/L NaCl solution (pH 2.3), both without and with 0.8 mol/L HAc (Figure 1, Figure 8, Figure 9 and Figure 10 and Table 1 and Table 5), were compared. In the case of 0.8 mol/L HAc, the *E*_corr_ of 304 SS clearly shifts positively (−213 mV to −134 mV), while both *I*_corr_ and *I*_pass_ increase significantly (0.456 μA/cm^−2^ to 2.124 μA/cm^−2^, 0.659 μA/cm^−2^ to 4.05 μA/cm^−2^, respectively), which reveals the promoting action of HAc on the uniform corrosion of 304 SS. This confirms previous studies in the literature that as a weak acid, the presence of HAc increases the corrosivity of a solution, and the corrosion rate is significantly higher when compared to strong acids at the same pH [13,49]. It was reported that acetic acid is not involved in a charge transfer process directly; its main contribution is in buffering the surface hydrogen ion concentration, thereby enhancing the limiting current and hence increasing the corrosion rate so that the corrosion potential shifts toward a more positive direction [49]. In addition, the dissociated acetate ions can adsorb onto the steel surface, participating in a dissolution reaction with Fe and Cr atoms, forming a soluble compound containing acetate, which has a higher solubility constant (*K*_SP_ = 10^−14^) than that of Fe(OH)_2_ (*K*_SP_ = 8 × 10^−16^) [1,15,28]. This will weaken the passivity of the film to some extent and enhance the uniform corrosion. Regarding the pitting corrosion, in a solution with 0.8 mol/L HAc, both the *E*_m_ and *E*_b_ of 304 SS move negatively, from 138 mV to 98.7 mV and from 348 mV to 336 mV, respectively, demonstrating that in the presence of HAc, both metastable pits and stable pits occur easily at relatively negative potentials. The average nucleation number of metastable pits decreased from 22 (without HAc) to 8 (with 0.8 mol/L HAc), indicating the significant inhibitory effect of HAc on the pit nucleation number. As discussed above, HAc can adsorb competitively with chloride ions at the active sites on the 304 SS surface [30], resulting in the blockage of the active sites, thereby reducing the number of initiation pits. Figure 10 shows a comparison of the average growth rate of metastable pits of 304 SS in two solutions (pH 2.3). It can be seen that the growth rate of metastable pits in the presence of HAc is a little higher than that without HAc, indicating that HAc has a certain facilitating action on the growth process of the metastable pits. This is probably because the dissociation of acetic acid is acting as an additional source of hydrogen ions. Some of the hydrogen ions could migrate into the metastable pits and accelerate the dissolution of metal inside the pits.

Pitting corrosion is a complicated process with stochastic characteristics. The metastable pitting corrosion behaviors of stainless steels in acetic acid with chloride ions could be influenced by many factors, such as the stability of the passive film, solution pH, the concentration of acetic acid and its dissociation degree, the adsorption of acetic acid, and the characteristics of the reaction products. To clarify the synthetic actions of all the above influencing factors, much research still needs to be carried out to understand and describe the process and the accompanying mechanisms.

## 4. Conclusions

(1) Adding a certain amount of HAc to the 0.6 mol/L NaCl solution had an obvious promoting effect on the uniform corrosion of 304 SS, due to the decreasing pH of the solution by the HAc. With an increase in HAc concentration, the corrosion rate showed a decreasing tendency, which might be due to the adsorption of HAc on the steel surface.
(2) The effects of HAc on the pitting corrosion of 304 SS are demonstrated as follows. On one hand, the metastable pits and the stable pits occur at relatively negative potentials (with the decreasing of *E*_m_ and *E*_b_) in the presence of HAc, while the growth process of metastable pits is promoted and easily transformed into bigger stable pits. On the other hand, the number of metastable pits is relatively lower.
(3) The addition of HAc causes a decrease in the ratios of Fe^3+^/Fe^2+^ and Cr_2_O_3_/Cr(OH)_3_ and, thereby, the decreased stability of the passive film. The metastable pitting corrosion of 304 SS in the mixed solution of HAc and NaCl is influenced by the synthetic actions of solution pH, acetic acid concentration and the nature of the passive film.

## Figures and Tables

**Table 1:** Parameters from the polarization curves of 304 SS in 0.6 mol/L NaCl solutions with HAc.

*C*_HAc_ (mol/L)	pH	*E*_corr_ (mV_SCE_)	*I*_corr_ (μA/cm^2^)	*I*_pass_ (μA/cm^2^)	*E*_m_ (mV_SCE_)	*E*_b_ (V_SCE_)	*E*_b_ − *E*_corr_ (V_SCE_)
0	7.2	−195	0.145	0.346	168	464	659
0.8	2.3	−134	2.124	4.050	98.7	336	470
1.6	2.2	−105	1.922	3.220	70.2	284	319
3.2	2.1	−98	1.022	2.040	−56.4	207	305

**Table 2:** EDS results of the metastable pits of 304 SS in 0.6 mol/L NaCl solutions with HAc.

C_HAc_ (mol/L)	Location	Si (at %)	P (at %)	S (at %)	Cr (at %)	Mn (at %)	Ca (at %)	Fe (at %)
0	1: Matrix	0.34	0.07	0.01	18.91	1.37	-	72.18
	2: Inside the pit	0.15	0.24	0.27	20.90	3.21	1.32	67.97
	3: Edge of the pit	0.53	0.77	0.26	19.13	2.05	1.21	65.30
0.8	1: Matrix	0.39	-	-	18.02	1.34	-	67.47
	2: Inside the pit	0.52	-	1.09	18.65	2.44	-	61.83
	3: Edge of the pit	0.82	0.36	0.85	15.69	0.72	-	59.16
1.6	1: Matrix	0.48	-	-	17.83	1.39	-	67.33
	2: Inside the pit	0.06	0.56	-	21.29	3.07	1.81	66.06
	3: Edge of the pit	0.44	-	-	17.63	1.40	-	66.86
3.2	1: Matrix	0.58	-	-	18.07	1.09	-	67.68
	2: Inside the pit	0.12	-	-	20.34	2.78	-	67.18
	3: Edge of the pit	0.84	-	-	16.01	0.94	0.36	59.20

**Table 3:** Amounts of H^+^, Ac^-^ and HAc in 0.6 mol/L NaCl solutions, with different concentrations of HAc.

Concentration of HAc Addition	[H^+^] (mol/L)	[Ac^-^] (mol/L)	[Undissociated HAc] (mol/L)
0.8 mol/L HAc (pH 2.3)	10^−2.3^	0.00243	0.79757
1.6 mol/L HAc (pH 2.2)	10^−2.2^	0.00442	1.59557
3.2 mol/L HAc (pH 2.1)	10^−2.1^	0.00703	3.19296

**Table 4:** The ratios of Fe^3+^/Fe^2+^ and Cr_2_O_3_/Cr(OH)_3_, calculated from the XPS spectra in the solutions with HAc.

HAc (mol/L)	0	0.8	1.6	3.2
Fe^3+^/Fe^2+^	1.85	0	0.56	0.72
Cr_2_O_3_/Cr(OH)_3_	1.45	1.28	1.22	1.22

**Table 5:** The parameters from the polarization curves of 304 SS in 0.6 mol/L NaCl solutions.

pH	*E*_corr_ (mV_SCE_)	*I*_corr_ (μA/cm^−2^)	*I*_pass_ (μA/cm^−2^)	*E*_m_ (mV_SCE_)	*E*_b_ (mV_SCE_)	*E*_b_-*E*_m_ (mV_SCE_)
7.2	−195	0.145	0.346	168	464	296
2.3	−213	0.456	0.659	138	348	210

## Data Availability

The raw/processed data required to reproduce these findings cannot be shared at this time as the data also forms part of an ongoing study.
